# Causal Association of Thyroid Signaling with C-Reactive Protein: A Bidirectional Mendelian Randomization

**DOI:** 10.1155/2022/8954606

**Published:** 2022-08-13

**Authors:** Tingting Li, Haigang Geng, Yuquan Wang, Zhaorong Wu, Siqian Yang, Yue-Qing Hu

**Affiliations:** ^1^State Key Laboratory of Genetic Engineering, Human Phenome Institute, Institute of Biostatistics, School of Life Sciences, Fudan University, Shanghai, China; ^2^Department of Gastrointestinal Surgery, Renji Hospital, School of Medicine, Shanghai Jiao Tong University, Shanghai, China; ^3^Department of Interventional Oncology, Renji Hospital, School of Medicine, Shanghai Jiao Tong University, Shanghai, China; ^4^Shanghai Center for Mathematical Sciences, Fudan University, Shanghai, China

## Abstract

**Methods:**

Based on the latest genome-wide association study summary data, bidirectional two-sample Mendelian randomization (MR) was employed to detect the causal relationship and effect direction between TSH, fT4, and CRP. Furthermore, in view of obesity being an important risk factor of CVD, obesity trait waist-hip ratio (WHR) and body mass index (BMI) were treated as the research objects in MR analyses for exploring the causal effects of TSH and fT4 on them, respectively.

**Results:**

Genetically increased CRP was associated with increased TSH (*β* = −0.02, *P* = 0.011) and with increased fT4 (*β* = 0.043, *P* = 0.001), respectively, but there was no evidence that TSH or fT4 could affect CRP. In further analyses, genetically increased TSH was associated with decreased WHR (*β* = −0.02, *P* = 3.99*e* − 4). Genetically increased WHR was associated with decreased fT4 (*β* = −0.081, *P* = 0.002). Genetically increased BMI was associated with increased TSH (*β* = 0.03, *P* = 0.028) and with decreased fT4 (*β* = −0.078, *P* = 1.05*e* − 4). Causal associations of WHR and BMI with thyroid signaling were not supported by weighted median analysis in sensitivity analyses.

**Conclusion:**

TSH and fT4 were increased due to the higher genetically predicted CRP. WHR was decreased due to the higher genetically predicted TSH. These findings will provide reference for the prevention and treatment of inflammation and metabolic syndrome.

## 1. Introduction

Subclinical thyroid disease is a common public health issue. In hypothyroidism due to thyroid dysfunction, serum thyroid-stimulating hormone (TSH) levels are appropriately elevated while serum free thyroxine (fT4) levels are within normal range [1, 2]. Subclinical hypothyroidism affects up to 10% of the adult population [3]. A lot of previous studies showed variation in TSH or fT4 may increase the risk of future cardiovascular diseases (CVD) [4–6]. Recently, many investigations indicated that even in normal thyroid function individuals, variation in TSH and fT4 was associated with an increased risk of CVD and metabolic diseases [7–9], including obesity [1]. Therefore, it is important and urgent to pay more attention to TSH and fT4.

C-reactive protein (CRP) is an acute-phase inflammatory protein, which has been traditionally utilized as a clinical marker of inflammation, infection, and tissue damage [10]. Generally, CRP exhibits elevated expression during inflammatory disorders, such as CVD, rheumatoid arthritis, and some acute or chronic infection [11, 12]. Recently, research outputs showed that minor CRP elevation could contribute to an increased future risk of major cardiovascular events [13, 14]. In addition, there was growing evidence that elevated CRP levels are associated with cancer disease risk [15, 16]. Hence, CRP measurements have potential utility as a clinical tool in assessing disease status and progression, including CVD, some infections, and cancer. Therefore, with the important role of CRP, more studies are needed to understand the complex mechanism of CRP production.

Existing literature demonstrated that subclinical hypothyroidism may be associated with elevated high-sensitive CRP, although the clinical implications were uncertain [17–19]. Some researchers found that there was a significant positive correlation between TSH and CRP [17, 20]. Meanwhile, a Brazilian longitudinal study of adult health also investigated the association between TSH and CRP, but this study showed that TSH was not associated with CRP because of the existence of confounders [21]. So, it was controversial about the relationship between thyroid signaling and CRP. Similarly, there were researches investigating the association of fT4 with CRP [18, 22, 23], and their conclusions were also controversial. Therefore, the association between thyroid signaling and CRP is hard to uncover.

Furthermore, as an important public health problem, obesity is also an important risk factor for CVD [11]. Previous investigations revealed a significant relationship between CRP and obesity [24–26]. In obese and overweight adults, CRP levels are significantly increased [27]. So it is meaningful to study obesity traits, such as waist-hip ratio (WHR) and body mass index (BMI).

Correlation describes whether two variables “go together.” However, the fact that two variables change together does not necessarily mean that we know whether one variable causes the other to change or vice versa [28]. Therefore, it is necessary to study causal association. To this end, one powerful method is Mendelian randomization (MR) [29], which uses genetic variants as instrumental variants (IVs) and has been widely used [30]. MR can minimize the influence of confounding factors on the causal association between two variables, exposure and outcome. Note both individual data and publicly available genome-wide association study (GWAS) summary statistics are applicable in MR analyses. Moreover, bidirectional two-sample MR can explore the nature and direction of the links between them.

To date, as far as we know, no studies investigated the causal associations of TSH and fT4 levels with CRP levels. In this paper, we studied the causal association between thyroid signaling and CRP level. To further detect the possible causes of CVD, we also studied two obesity traits, WHR and BMI. For this, we utilized summary data from the latest and largest GWASs [31–33] and inferred causality in bidirectional two-sample MR analyses.

## 2. Materials and Methods

### 2.1. Data Sources

The first is the source of summary data related to thyroid signaling. Summary data for TSH within reference range were obtained from a GWAS meta-analysis that is the largest GWAS on thyroid function to date, including 120000 subjects, with more than 22 million single nucleotide polymorphisms (SNPs) [31]. These data are accessed through the GWAS Catalog (https://www.ebi.ac.uk/gwas). Summary data for fT4 within reference range were obtained from a GWAS meta-analysis in up to 72167 individuals with 8 million SNPs [32], which can be downloaded on dbGaP website under the accession number phs000930 (https://www.ncbi.nlm.nih.gov/gap).

Second is the source of summary data on inflammatory factor CRP. Summary data for CRP were obtained from a GWAS meta-analysis which is the largest data set on inflammatory factors lately, including 49839 subjects (CRP: mean = 4.114 (SD = 4.836)) [33]. These summary data can be available through the GWAS Catalog (https://www.ebi.ac.uk/gwas). Summary data for WHR were obtained from a GWAS meta-analysis in 694649 individuals of European ancestry with 2.7 million SNPs combining UK Biobank and GIANT [34]. Summary data for BMI were obtained from a GWAS meta-analysis which included about 700000 participants of European ancestry with 2.3 million SNPs from GIANT [35].

### 2.2. Two-Sample MR

**Figure 1:**
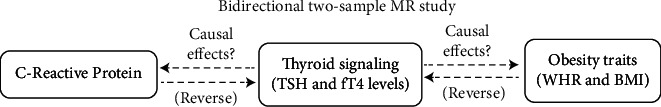
Schematic diagram. Bidirectional two-sample MR approach based on the summary level data from large scale meta-analyses of the GWASs was used to investigate the causal relationships between thyroid signaling and CRP. Further bidirectional two-sample MR approach was used to investigate the causal relationships between thyroid signaling and obesity traits. All data sets used in this study are publicly available at the GWAS Catalog, dbGaP, and the GIANT websites. TSH: thyroid-stimulating hormone; fT4: free thyroxine; GWAS: genome-wide association study; CRP: C-reactive protein; MR: Mendelian randomization; WHR: waist-hip ratio; BMI: body mass index.

We conducted bidirectional two-sample MR analyses using data published by GWAS (Figure 1). Because the data is public, there is no need of ethical review.

### 2.3. Selection of SNPs

Based on the GWAS results [31–33] on TSH, fT4, and CRP, we used independent SNPs which are strongly associated at a genome-wide significant level (*P* < 5 × 10^−8^) with TSH, fT4, and CRP, respectively. The selected SNPs were used as IVs in using MR method.

### 2.4. Statistical Analysis

In order to avoid the estimator bias caused by weak IVs as much as possible, we calculated the *F* statistic (*F* = *β*_exposure_^2^/SE_exposure_^2^) as a measure of strength for each SNP. According to the existing literature, criterion of *F* ≥ 10 was adopted for screening strong IVs (*F* statistic was in 30.01–1231.188 for TSH, 30.25–455.33 for fT4, and 27.94–528.51 for CRP) [36]. The primary analysis used to examine the causality between exposure and outcome was inverse-variance weighted (IVW) method [37]. IVW method aggregated two or more IVs to minimize the variance of the weighted average, and the weight given to each IV was the inverse of the variance of the effect estimate [38]. Note that the estimated effect obtained by IVW may be biased, which may be due to the violation of one assumption of IV. Specifically, IVs and outcome are not only related through exposure but also directly related, which is termed as pleiotropy. We addressed the problem of pleiotropy in sensitivity analyses.

In sensitivity analyses, we assessed the robustness of IVW in two complementary sensitivity analyses with different assumptions about horizontal pleiotropy: weighted median (MR-Median) [38] and MR-Egger regression [39]. MR-Median yielded consistent causal effect estimates compared with IVW method. Egger intercept in MR-Egger represented the average horizontal pleiotropic effect across the IVs. We used *I*^2^ statistic and Cochran's *Q* test to quantify heterogeneity across all SNPs. If the results indicated the presence of horizontal pleiotropy or significant heterogeneity suggesting pleiotropy [40], we calculated individual *Q* statistic for each SNP, and SNPs were identified as potential pleiotropic variants if their individual *Q* statistics exceeded the 95th percentile of the chi-square distribution with one degree of freedom [41–43]. After excluding these potential pleiotropic IVs, the IVW, MR-Median, and MR-Egger methods were performed on the remaining IVs.

For the estimated causal effect of the exposure on the outcome, a *P* value of less than 0.05 was considered as statistically significant. Statistical analysis was performed with R package “MendelianRandomization” version 0.5.1 in R version 4.1.0.

## 3. Results

**Figure 2:**
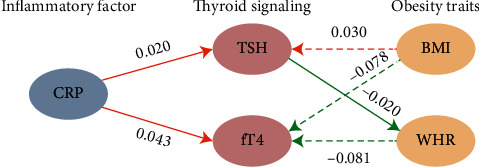
Causal effects between thyroid signaling, CRP, and obesity traits. TSH and fT4 are shown in pink ovals, CRP is shown in gray ovals, and BMI and WHR are shown in yellow ovals. The arrows' direction denotes causal direction. The solid line and the dotted line, respectively, indicate whether the causal relationship is robust or not. The red and green arrows denote positive and negative causal relationships, respectively, and the number beside each arrow is the causal effects. All causal relationships are significant at *P* ≤ 0.05.

Use the MR analysis method in previous sections to explore the causal relationships between thyroid signaling, CRP, and obesity traits. The MR-Egger intercepts were insignificant (*P* > 0.05) in all analyses. The result diagram is shown in Figure 2. The diagram showed whether there was a causal relationship between two subjects and showed the magnitudes and directions of the causal relationships. All causal relationships are significant at *P* ≤ 0.05.

### 3.1. Causal Relationships between Thyroid Signaling and CRP

**Figure 3:**
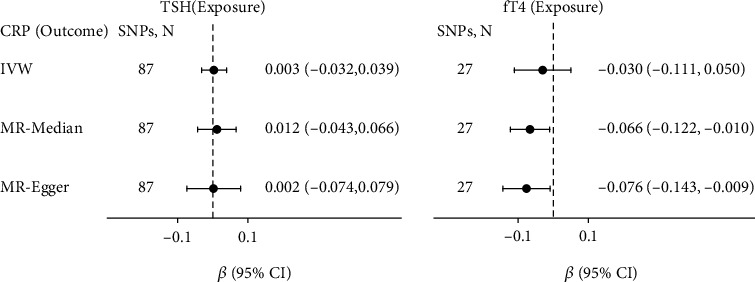
Causal effects of variation in TSH and fT4 levels on CRP. Presented *β*s and CIs (horizontal lines and their corresponding numerical interval form on the right side) correspond to the effects of a one SD change in TSH or fT4 levels on the outcome CRP levels. The results of MR analyses using various analysis methods (IVW, MR-Median, MR-Egger) are presented for comparison. The number of SNPs indicates the number of genetic variants used as instrument variables for MR analysis. MR: Mendelian randomization; SNPs: single nucleotide polymorphisms; CI: confidence interval; IVW: inverse-variance weighted; MR-Median: weighted median method; MR-Egger: Egger regression.

The results of MR analyses between genetically predicted TSH and fT4 levels (exposure) and CRP levels (outcome) are presented in Figure 3. 87 SNPs were in consideration when we investigated the causal association between TSH and CRP and 30 SNPs for fT4 and CRP. Based on this analysis, we found neither serum TSH nor fT4 levels could cause changes in CRP (TSH: *β* = 0.003, 95% CI = −0.032–0.039, *P* = 0.856; fT4: *β* = 0.003, 95% CI = −0.084–0.089, *P* = 0.953) (also, see details in Supplementary Table S1).

**Figure 4:**
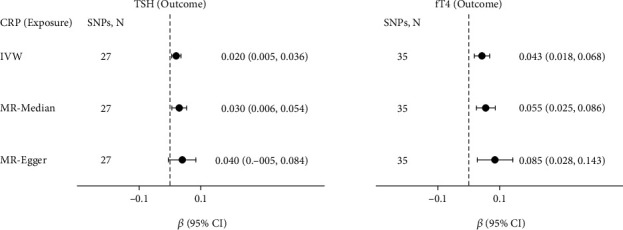
Causal effects of variation in CRP levels on TSH and fT4. Presented *β*s and CIs (horizontal lines and their corresponding numerical interval form on the right side) correspond to the effects of a one SD change in CRP levels on the outcome TSH or fT4 levels. The results of MR analyses using various analysis methods (IVW, MR-Median, MR-Egger) are presented for comparison. The number of SNPs indicates the number of genetic variants used as instruments for MR analysis.

However, exchanging the exposure and outcome of interest in MR yielded different results (Figure 4). 27 SNPs were considered as IVs when we investigated the causal association between CRP and TSH. There was some evidence that higher CRP levels might cause higher TSH levels (*β* = 0.02, 95% CI = 0.005–0.036, *P* = 0.011), which was confirmed in sensitivity analyses using MR-Median method (see Supplementary Table S2).

For the association between CRP and fT4, 35 SNPs were taken as IVs as shown in Figure 4. There was some strong evidence that higher genetically predicted FT4 might cause higher CRP levels (*β* = 0.013, 95% CI = 0.018–0.068, *P* = 0.001), which was also in line with the results of sensitivity analyses using the MR-Median and MR-Egger method (see Supplementary Table S2).

### 3.2. Causal Relationships between Thyroid Signaling and Obesity Traits

**Figure 5:**
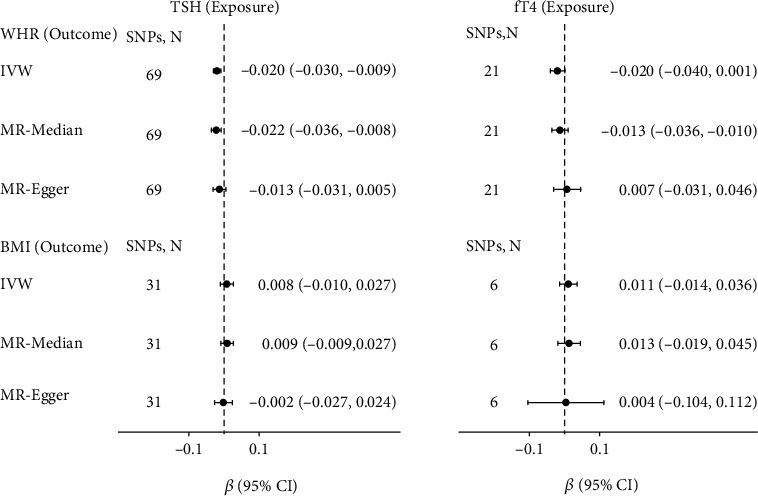
Causal effects of variation in TSH and fT4 levels on WHR and BMI. Presented *β*s and CIs (horizontal lines and their corresponding numerical interval form on the right side) correspond to the effects of a one SD change in TSH or fT4 levels on the outcome WHR or BMI levels. The results of MR analyses using various analysis methods (IVW, MR-Median, MR-Egger) are presented for comparison. The number of SNPs indicates the number of genetic variants used as instruments for MR analysis.

Due to elevated CRP levels in overweight and obese adults [27], we also wanted to know whether there exist causal associations between thyroid signaling and obesity traits. Therefore, we conducted MR analyses of TSH and fT4 on obesity traits, respectively (Figure 5). MR analyses showed higher genetically predicted TSH could cause decreased WHR levels (*β* = −0.02, 95% CI = −0.030–0.009, *P* = 3.99*e* − 4), and MR-Median also led to similar results (see Supplementary Table S3 for more information).

**Figure 6:**
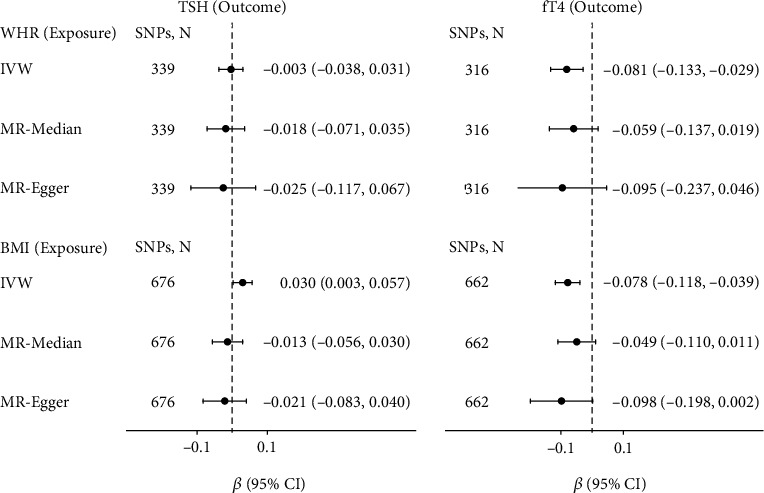
Causal effects of variation in WHR and BMI levels on TSH and fT4. Presented *β*s and CIs (horizontal lines and their corresponding numerical interval form on the right side) correspond to the effects of a one SD change in WHR or BMI levels on TSH or fT4 levels. The results of MR analyses using various analysis methods (IVW, MR-Median, MR-Egger) are presented for comparison. The number of SNPs indicates the number of genetic variants used as instruments for MR analysis.

For the association between obesity traits and thyroid signals (Figure 6), MR analyses showed higher genetically predicted WHR could cause decreased fT4 levels (*β* = −0.081, 95% CI = −0.133–-0.029, *P* = 0.002), and higher genetically predicted BMI could cause higher TSH levels (*β* = 0.030, 95% CI = 0.003–0.057, *P* = 0.028) and lower fT4 (*β* = 0.02, 95% CI = −0.118–0.068, *P* = 1.05*e* − 4). This causal relationship was not robust because it is not supported by MR-Median (see Supplementary Table S4).

## 4. Discussion

In this study, the bidirectional two-sample MR analyses between thyroid signaling (TSH and fT4) and CRP levels were accomplished based on the current largest GWAS summary statistics. We studied the causal relationships between TSH and fT4 levels and CRP levels and found TSH and fT4 levels could be affected by CRP, whereas TSH and fT4 levels could not affect CRP levels. Furthermore, we found some evidence that there were associations between obesity traits (BMI and WHR) and fT4 levels. We also found that TSH could be significantly affected by BMI.

CRP responds quickly to inflammatory processes and is utilized as one of the best inflammatory markers. Various research results showed that there was a significant positive correlation between TSH and CRP [17, 44, 45]. However, their underlying causality was still unclear. A prospective study indicated that patients with subclinical hypothyroidism had increased levels of signs of low-grade inflammation (CRP levels) [20, 46]. On the other hand, the conclusions of many studies were not consistent with this prospective study. For example, some authors believed that serum CRP was not significantly affected by the thyroid dysfunction's degree [47]. An observational study found that CRP was not correlated with fT4 and TSH [48]. These studies indicated that further evidence was needed to determine the causal link between TSH and fT4 levels and CRP levels. In this study, based on MR analysis results, we found that there was a causal association between CRP and thyroid signaling (TSH and fT4). TSH and fT4 levels could be positively affected by CRP levels, but not vice versa. The underlying cause of CRP affecting thyroid signaling is still unclear, possibly because severe inflammation may significantly affect the thyroid gland, leading to changes in thyroid signaling. Besides, we thought there were some potential effects of inflammation on deiodinase activity. Inflammation (elevated in CRP levels) which was related to infection or injury led to a reduction in deiodinase activity. This results in decreased conversion of fT4 to fT3, leading to high fT4 [17]. In the future, the causal relationship between CRP levels and TSH and fT4 levels may be confirmed with larger populations and more precise statistical methods.

Interestingly, in a Brazilian longitudinal study of adult health, obesity was considered as one of the most important confounders in the association study between TSH and CRP [21]. Some researches showed that CRP was correlated with obesity and the role of obesity in inflammation can not be ignored [49]. This promoted us to study further the obesity traits.

In the subsequent MR analyses, we found that increased TSH could cause decreased WHR. In reverse MR analyses, increased WHR and BMI could cause decrease in fT4, and increased BMI could cause increase in TSH. Previous literature showed that lower fT4 was consistently associated with obesity in healthy euthyroid people [50, 51]. One research indicated that serum fT4 levels were negatively correlated with BMI and serum TSH levels were positively correlated with WHR and BMI [52]. It was suggested that the increase in fT3 levels in obese people may be a compensatory mechanism for the fat accumulation increase [53]. In obese people, thyroxine 5-deiodinase increased activity, inducing the increased peripheral conversion of fT4 to fT3 [49, 54]. The lower fT4 in obese and overweight people might partially result from this cause. These were consistent with the results of our MR study. Conclusion of a recent MR analysis was also consistent with our study; i.e., genetically predicted BMI was inversely associated with fT4 levels [55].

Another MR analysis pointed out that TSH could be significantly elevated by the genetically driven BMI, while fT4 could not be affected by BMI [56]. Notice in our study, we used the latest and largest GWAS summary data, where fT4 cohorts included nearly 70000 participants. Moreover, we performed sensitivity analyses to exclude pleiotropic and heterogeneous IVs, because these heterogenous SNPs could partially result in bias in MR analysis. In our analysis, TSH and fT4 both could be affected by BMI.

Advantages of this study design were that (1) GWAS data were freely available obtained from the largest recent GWAS on TSH, fT4, and CRP, respectively; (2) sensitivity analyses were performed in order to reduce potential bias resulting from potential pleiotropic and heterogeneous IVs. It is the first time to reach a conclusion based on MR analysis that higher genetically predicted CRP may induce an increase in TSH and fT4. However, this study has certain limitations. (1) Due to the accession of the public databases, we used people of diverse ancestry for CRP and people of European ancestry for thyroid signaling and obesity traits. MR analysis for population-stratification and other populations should be considered if related data can be available; (2) generally speaking, thyroid function is sex-specific; due to the limitation of TSH and fT4 summary data, we did not perform the sex-specific MR analyses.

Taken together, the bidirectional MR study demonstrated that higher TSH and fT4 levels were causally affected by higher CRP levels, but not vice versa. Further MR analyses provided evidence that higher obesity traits could cause lower fT4 and higher BMI could cause higher TSH.

## Data Availability

All data generated or analyzed during this study are included in this published article or in the data repositories listed in References. Summary statistic data for genetic associations with thyroid signaling have been contributed by the thyroid GWAS meta-analysis of Hunt and the ThyroidOmics consortium. Summary statistic data for genetic association with body mass index and waist-hip ratio have been contributed by the GIANT consortium and MEGASTROKE consortium. Summary statistic data for genetic association with C-reactive protein have been contributed by the Population Architecture Using Genomics and Epidemiology study.

## Supplementary Materials

Supplementary Materials Supplementary Table S1: results of Mendelian randomization analyses between genetically predicted thyroid-stimulating hormone (TSH) and free thyroid hormone (fT4) levels (exposure) and C-reactive protein (CRP) levels (outcome). Supplementary Table S2: results of Mendelian randomization analyses between genetically predicted CRP levels (exposure) and TSH and fT4 levels (outcome). Supplementary Table S3: results of Mendelian randomization analyses between genetically predicted TSH and fT4 levels (exposure) and obesity traits (outcome). Supplementary Table S4: results of Mendelian randomization analyses between genetically predicted obesity traits (exposure) and TSH and fT4 levels (outcome).
