# Supplementary material for: Causal Association of Thyroid Signaling with C-Reactive Protein: A Bidirectional Mendelian Randomization
Source: Comput Math Methods Med. 2022 Aug 13;2022:8954606. doi: 10.1155/2022/8954606 (PMC9392607; doi:10.1155/2022/8954606)
Supplement: Supplementary Materials — Supplementary Table S1: results of Mendelian randomization analyses between genetically predicted thyroid-stimulating hormone (TSH) and free thyroid hormone (fT4) levels (exposure) and C-reactive protein (CRP) levels (outcome). Supplementary Table S2: results of Mendelian randomization analyses between genetically predicted CRP levels (exposure) and TSH and fT4 levels (outcome). Supplementary Table S3: results of Mendelian randomization analyses between genetically predicted TSH and fT4 levels (exposure) and obesity traits (outcome). Supplementary Table S4: results of Mendelian randomization analyses between genetically predicted obesity traits (exposure) and TSH and fT4 levels (outcome). [file 8954606.f1.docx]

**Supplementary Table S1**: Results of Mendelian randomization analyses between genetically predicted TSH and fT4 levels (exposures) and CRP levels (outcome) using three MR methods

|  | TSH (exposure)~CRP | | | fT4 (exposure)~CRP | | |
| --- | --- | --- | --- | --- | --- | --- |
| Method | beta | SE | P | beta | SE | P |
| IVW | 0.003 | 0.018 | 0.856 | 0.003 | 0.044 | 0.953 |
| MR-Median | 0.012 | 0.028 | 0.679 | 0.028 | 0.045 | 0.528 |
| MR-Egger | 0.002 | 0.039 | 0.949 | 0.067 | 0.084 | 0.427 |
| (Egger Intercept) | <0.001 | 0.002 | 0.928 | -0.005 | 0.005 | 0.4 |

Egger intercept in MR-Egger method represent estimated pleiotropy information in analysis. When we investigated the causal relationship between TSH (exposure) and CRP (outcome), there were 87 SNPs. When we investigated fT4 (exposure) and CRP (outcome), there were 30 SNPs.

Abbreviations: IVW: inverse variant weighted method; MR: Mendelian randomization; SNP: single nucleotide polymorphism; MR-Median: weighted median method; MR-Egger: Egger regression method; TSH: thyroid-stimulating hormone; fT4: free thyroid hormone; CRP: C-reactive protein.

**Supplementary Table S2:** Results of Mendelian randomization analyses between genetically predicted CRP levels (exposures) and TSH and fT4 levels (outcome) using three MR methods

|  | CRP (exposure)~TSH | | | CRP (exposure)~fT4 | | |
| --- | --- | --- | --- | --- | --- | --- |
| Method | beta | SE | P | beta | SE | P |
| IVW | 0.02 | 0.008 | **0.011** | 0.043 | 0.013 | **0.001** |
| MR-Median | 0.03 | 0.012 | **0.014** | 0.055 | 0.016 | **3.60e-4** |
| MR-Egger | 0.04 | 0.023 | 0.081 | 0.085 | 0.03 | **0.004** |
| (Egger Intercept) | -0.003 | 0.003 | 0.34 | -0.006 | 0.004 | 0.123 |

Egger intercept in MR-Egger method represent estimated pleiotropy information in analysis. When we investigated the causal relationship between CRP (exposure) and TSH (outcome), there were 27 SNPs. When we investigated CRP (exposure) and fT4 (outcome), there were 35 SNPs.

**Supplementary Table S3**: Results of Mendelian randomization analyses between genetically predicted TSH and fT4 levels (exposures) and obesity traits (outcomes) using three MR methods

|  | TSH (exposures) | | | fT4 (exposures) | | |
| --- | --- | --- | --- | --- | --- | --- |
| WHR (outcomes) |  |  |  |  |  |  |
| Method | Beta | SE | P | beta | SE | P |
| IVW | -0.020 | 0.006 | **3.99e-4** | -0.02 | 0.01 | 0.061 |
| MR-Median | -0.020 | 0.007 | **0.002** | -0.013 | 0.012 | 0.269 |
| MR-Egger | -0.013 | 0.009 | 0.15 | 0.007 | 0.02 | 0.711 |
| (Egger Intercept) | 0 | 0.001 | 0.419 | -0.002 | 0.001 | 0.139 |
| BMI (outcomes) |  | | |  | | |
| IVW | 0.008 | 0.009 | 0.371 | 0.011 | 0.013 | 0.374 |
| MR-Median | 0.009 | 0.009 | 0.348 | 0.013 | 0.016 | 0.418 |
| MR-Egger | -0.002 | 0.013 | 0.884 | 0.004 | 0.055 | 0.947 |
| (Egger Intercept) | 0.001 | 0.001 | 0.433 | 0.001 | 0.003 | 0.8 |

Intercept in MR-Egger method represent estimated pleiotropy information in analysis. When we investigated the causal relationship between TSH (exposure) and WHR (outcome), there were 69 SNPs. When we investigated fT4 (exposure) and WHR (outcome), there were 21 SNPs. When we investigated the causal relationship between TSH (exposure) and BMI (outcome), there were 31 SNPs. When we investigated fT4 (exposure) and BMI (outcome), there were 6 SNPs.

**Supplementary Table S4:** Results of Mendelian randomization analyses between genetically predicted obesity traits (exposures) and TSH and fT4 levels (outcome) using three MR methods

|  | TSH (outcome) | | | fT4 (outcome) | | |
| --- | --- | --- | --- | --- | --- | --- |
| WHR (exposure) |  |  |  |  |  |  |
| Method | beta | SE | P | beta | SE | P |
| IVW | -0.003 | 0.018 | 0.846 | -0.081 | 0.027 | **0.002** |
| MR-Median | -0.018 | 0.027 | 0.508 | -0.059 | 0.04 | 0.135 |
| MR-Egger | -0.025 | 0.047 | 0.595 | -0.095 | 0.072 | 0.185 |
| (Egger Intercept) | 0 | 0.001 | 0.663 | 0 | 0.001 | 0.88 |
| BMI (exposure) |  | | |  | | |
| IVW | 0.03 | 0.014 | **0.028** | -0.078 | 0.02 | **1.05e-4** |
| MR-Median | -0.013 | 0.022 | 0.553 | -0.049 | 0.031 | 0.11 |
| MR-Egger | -0.021 | 0.031 | 0.498 | -0.098 | 0.051 | 0.055 |
| (Egger Intercept) | 0.001 | 0.001 | 0.069 | 0 | 0.001 | 0.714 |

Intercept in MR-Egger method represent estimated pleiotropy information in analysis. When we investigated the causal relationship between WHR (exposure) and TSH (outcome), there were 339 SNPs. When we investigated WHR (exposure) and fT4 (outcome), there were 316 SNPs. When we investigated the causal relationship between BMI (exposure) and TSH (outcome), there were 676 SNPs. When we investigated BMI (exposure) and fT4 (outcome), there were 662 SNPs.
